# Does the Fat Tailed Damara Ovine Breed Have a Distinct Lipid Metabolism Leading to a High Concentration of Branched Chain Fatty Acids in Tissues?

**DOI:** 10.1371/journal.pone.0077313

**Published:** 2013-10-18

**Authors:** Susana P. Alves, Rui J. B. Bessa, Mário A. G. Quaresma, Tanya Kilminster, Tim Scanlon, Chris Oldham, John Milton, Johan Greeff, André M. Almeida

**Affiliations:** 1 CIISA – Centro Interdisciplinar de Investigação em Sanidade Animal, Faculdade de Medicina Veterinária/UTL, Lisboa, Portugal; 2 DAFWA – Department of Agriculture and Food Western Australia, Perth, Western Australia, Australia; 3 Faculty of Natural and Agricultural Sciences, UWA – University of Western Australia, Crawley, Western Australia, Australia; 4 Biotrop, IICT – Instituto de Investigação Científica Tropical, Lisboa, Portugal; 5 IBET – Instituto de Biologia Experimental e Tecnológica, Oeiras, Portugal; 6 BCV, ITQB/UNL – Instituto de Tecnologia Química e Biológica, Oeiras, Portugal; Max Delbrueck Center for Molecular Medicine, Germany

## Abstract

Fat tailed sheep breeds are known for their adaptation to nutritional stress, among other harsh production conditions. Damara sheep, native to Southern Africa, have recently been exported to other areas of the world, particularly Australia, aiming to produce lamb in semi-arid regions. Damaras have a unique hanging fat tail, a fat depot able to be mobilized under nutritional stress. In this article we perform an in-depth characterization of the fatty acid profiles of the fat tail in underfed and control Damara rams. Profiles were very similar between experimental groups, with the exception of palmitic acid (16:0) that was lower (P = 0.014) in underfed animals. However, the most striking result was the very high proportions of non-terminal branched chain fatty acids found in the fat tail adipose tissue, as well as the gastrocnemius muscle of Damara rams. The muscle of Dorper and Merino rams used in the same experiment did not present non-terminal branched chain fatty acids, suggesting that Damara rams have a unique lipid metabolism. Herein, we interpret this trait relating it to a higher ability of Damara sheep to digest fibrous fodder and to putative differences in the propionate metabolism by comparison to other sheep breeds.

## Introduction

**Figure 1:**
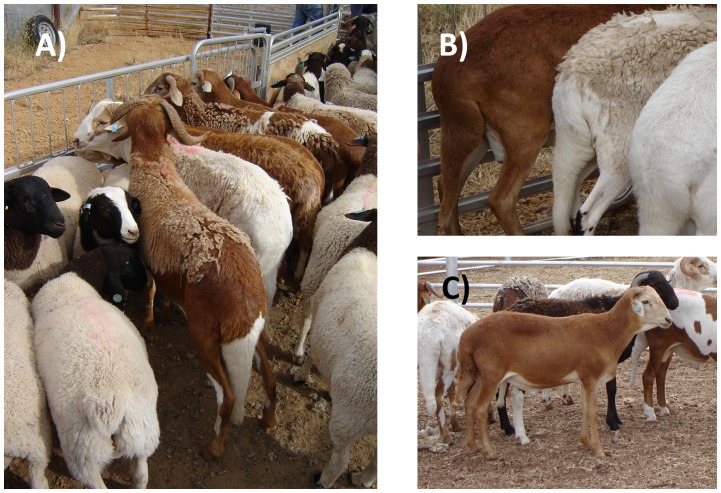
Damara fat tailed sheep. A) Damara, Dorper and Australian Merino rams at the initial stages of the feeding trial. Notice the characteristic hanging fat tail of the ram up front. B) Several aspects of the fat tail in several of the Damara rams used in this trial. C) In the first plane, brown Damara ram.

Fat tail and fat rump sheep are characteristic of semi-arid environments and commonly found across vast areas of the globe: Eastern and Southern Africa, the steppes of Central Asia, as well as numerous countries in the Middle-East. The Damara (see Figure 1) is one of such fat tailed sheep breeds characterized by a large body frame and a shedding hair coat. Damaras originated from Southern Angola and Namibia and were later selected in South Africa [1]. Fat tailed sheep are reputed for being highly resilient to harsh environmental conditions such as diseases, parasites, water scarcity or seasonal weight loss, but experimental support for such claims are scarce. Recently, the resistance to feed restriction of male rams of three breeds present in Australia (Merino, Dorper and Damara) was evaluated [2]. Samples of tail adipose tissue of the Damara rams used in that experiment were collected and analysed for fatty acid composition. To our knowledge, no information of fatty acid composition of adipose tissue from Damara tails is available in scientific literature. Moreover, fatty acid composition of adipose tissue of fat tails of other breeds is generally limited to the major fatty acids [3]–[7] and more detailed fatty acid profiles are scarce [8].

As the tail fat depot is naturally mobilized (increased lipolysis and reduced deposition) in animals submitted to feed restriction, we hypothesized that this would also induce significant changes in fatty acid composition. Thus, our aim was to report the fatty acid composition of adipose tissue of Damara tails and relate it to feed restriction. However, we extended the fatty acid analysis to muscle samples in order to explain the unusual abundance of odd and branched chain fatty acids (BCFA) found in the Damara tail fat.

## Materials and Methods

### Animal Experiment

All animal work was conducted according to relevant international guidelines (European Union procedures on animal experimentation – Directive 2010/63/EU) that regulate the use of production animals in animal experimentation. These define that in the case of experiments carried out under standard production conditions, no approval from an ethics committee is required. Nevertheless, this experiment was conducted with the approval of the Ethics Committee of the Department of Agriculture and Food Western Australia (DAFWA, Perth, WA, Australia) registered as process 07ME06. The entire trial was conducted under the supervision of the competent veterinary authority in the State of Western Australia. Additionally, author AM Almeida holds a FELASA (Federation of European Laboratory Animal Society Associations) grade C certificate that enables designing and carrying out animal experimentation under European Union regulations. Animal management, handling, transport and sacrifice were all conducted replicating approved standard commercial practices in the Commonwealth of Australia and in the State of Western Australia. Animals included in this experiment were therefore subjected to the same welfare conditions as production animals in farms and stations.

The detailed information on animals, management, diet, experimental design and productive and carcass traits have been previously reported [2], [9]. Briefly, twelve 6 month old ram lambs of the Dorper, Damara and Australian Merino breeds were individually fed on the same diet at different feeding levels for 42 d in order that one group was allowed to grow 100 g/head/d and the other to lose 100 g/head/d. Here we focused on samples from the fat tail and gastrocnemius muscle of Damara breed, using muscle samples from the other breeds to verify the presence of BCFA.

All animals were exclusively fed on the same commercial roughage-based feed pellet (Macco 101, Macco Feeds, Williams, WA, Australia), with 9.3 MJ/kg dry matter (DM) of metabolized energy (ME), 115 g/kg DM of crude protein (CP) and 291 g/kg of acid detergent fiber (ADF). The fatty acid content of the feed pellet was 15.1 g/kg DM, and the major fatty acids (in percentage of total fatty acids) were 18:1 *cis*-9 (32.5%), 16:0 (30.0%), 18:2 n-6 (27.9%), 18:0 (4.3%) and 18:3 n-3 (2.1%).

At the trial completion animals were transported to a licensed commercial abattoir (Tammin abattoir, Tammin, WA, Australia) and slaughtered following standard commercial practices. Briefly, animals were stunned and carcasses were decapitated, skinned and eviscerated and the fat tail was immediately excised and weighted. Fat tail and the gastrocnemius muscle were sampled (40–70 g) and preserved at −80°C in liquid nitrogen until further analysis.

### Fatty acid analysis

Lipids from lyophilized fat tail and muscle tissues were extracted according to the procedure of Folch et al. [10], but using dichloromethane and methanol (2:1, v/v) instead of chloroform: methanol. The fatty acid methyl esters (FAME) were prepared from the lipid extracts with sodium methoxide in methanol followed by hydrochloric acid in methanol (1:1, v/v). The methyl nonadecanoate (19:0) was used as internal standard. A few muscle samples of the FAME extracts from Damara, Merino and Dorper ram lambs, were hydrogenated using Adam´s catalyst (platinum dioxide) under hydrogen at 50°C for 1 hour in order to reveal more clearly the presence of the branched chain fatty acids.

Samples were analyzed using a gas chromatograph HP6890A (Hewlett-Packard, Avondale, PA, USA), equipped with a flame-ionization detector (GLC-FID) and a CP-Sil 88 capillary column (100 m; 0.25 mm i.d.; 0.20 µm film thickness; Agilent Technologies Inc., Santa Clara, CA, USA). The column oven temperature were as follows: initial temperature of 100°C was held for 15 min, increased to 150°C at a rate of 10°C/min and held for 5 min, then increased to 158°C at 1°C/min and held for 30 min, and finally increased to 200°C at a rate of 1°C/min and maintained for 65 min. Helium was used as carrier gas, and the injector and detector temperatures were 250 and 280°C, respectively. Identification of FAME was achieved by comparison of the FAME retention times with those of authentic standards (FAME mix 37 components from Supelco Inc., Bellefont, PA, USA). Additional characterization of the FAME, particularly the branched chain structures, was achieved by electron impact (EI) mass spectrometry using a Varian Saturn 2200 system (Varian Inc., Walnut Creek, CA, USA) equipped with the same capillary column and with the oven temperatures used for GLC-FID analysis. The mass spectra conditions were as follows: trap temperature, 150°C; manifold temperature, 45°C; transferline temperature, 220°C; EI ionization energy, 70eV; scan, 50–650 atomic mass units.

### Statistical analysis

Data were analyzed using Proc MIXED of SAS (SAS Inst., Cary, NC, USA) with a model that included the treatment (growth vs. restriction) as the single effect. Significance was declared at P<0.05.

## Results

The Damara animals submitted to feed restriction had lower (P<0.05) live weight and carcass weight and tended (P = 0.08) to had lower tail weight than animals allowed to grow (Table 1). Nevertheless, the tail weight expressed either as percentage of live weight or as percentage of carcass weight did not differ between experimental groups.

**Table 1:** Effect of feed restriction on the growth and fat tail in Damara ram lambs.

	Growth	Restricted	SEM	P
n	12	12		
Tail weight (g)	1103	830	105	0.080
Carcass weight (kg)	20.3	16.7	0.96	0.016
Tail (% of carcass)	5.63	4.94	0.581	0.412
Live slaughter weight	46.0	37.3	1.583	0.001
Tail (% live slaughter weight)	2.43	2.21	0.239	0.520

The fatty acid composition of tail fat expressed in percentage of total fatty acids is presented in Table 2. Animals submitted to feed restriction had significantly (P<0.05) lower palmitic acid (16:0) and tended (*P* = 0.063) to have higher oleic acid (18:1 *cis-*9) than animals allowed to grow. Besides this, only two other minor fatty acids (16:0–2Me and 18:3 *cis-*9, *trans-*11, *cis-*15) displayed significant differences between treatments. Thus, the fatty acid profile remained notably similar between treatments. The fatty acid profile of tail fat was dominated by 18:1 *cis-*9, 16:0 and 18:0 that together comprised 66.6% of total fatty acids. The biohydrogenation intermediates (18:1, 18:2 and 18:3 isomers excluding the 18:1 *cis-*9, 18:1 *cis*-11, 18:2 n-6 and 18:3 n-3) comprised 6.9% of total fatty acids. Notably, the sum of BCFA reached 8.8% of total fatty acids and 77% of these are not the common terminal branched chain (i.e iso and anteiso). Also, the sum of odd linear chain fatty acids reach 5.1%, and odd and BCFA together comprise 14% of total fatty acids.

**Table 2:** Effect of feed restriction in the fatty acid composition (% of total fatty acids) of the fat tail in Damara ram lambs.

Fatty Acid	Growth	SEM	Restricted	SEM	P value
N	12		11		
10:0	0.13	±0.016	0.12	±0.017	0.436
10:0–4Me/-6 Me	0.16	±0.019	0.12	±0.020	0.201
11:0	0.05	±0.004	0.04	±0.004	0.917
11:0–4 Me	0.11	±0.011	0.10	±0.012	0.669
11:0–4,8 Me	0.08	±0.010	0.08	±0.011	0.802
12:0	0.07	±0.005	0.08	±0.005	0.140
12:0–4 Me	0.20	±0.018	0.18	±0.019	0.276
12:0–8 Me	0.12	±0.009	0.11	±0.009	0.574
12:0–4,8 Me	0.19	±0.018	0.16	±0.019	0.250
13:0	0.09	±0.007	0.10	±0.007	0.482
13:0–6 Me	0.03	±0.003	0.03	±0.003	0.268
13:0–4 Me	0.18	±0.014	0.18	±0.015	0.864
iso-14:0	0.04	±0.002	0.05	±0.002	0.795
13:0–4,8 Me	0.11	±0.011	0.11	±0.011	0.944
14:0–2 Me	0.05	±0.008	0.06	±0.009	0.416
14:0	2.00	±0.102	2.01	±0.107	0.911
14:0–6 Me	0.36	±0.021	0.35	±0.022	0.771
14:0–8 Me	0.33	±0.019	0.33	±0.019	0.768
14:0–4 Me	0.68	±0.041	0.56	±0.044	0.074
14:0–10 Me	0.37	±0.023	0.37	±0.024	0.970
iso-15:0	0.48	±0.027	0.46	±0.028	0.667
anteiso-15:0	0.35	±0.016	0.35	±0.017	0.910
14:0–2,6 Me	0.14	±0.012	0.16	±0.013	0.240
14:1 *cis-*9	0.11	±0.012	0.09	±0.012	0.159
15:0	1.49	±0.068	1.50	±0.071	0.873
15:0–8 Me	0.41	±0.028	0.46	±0.029	0.236
15:0–4 Me	0.46	±0.032	0.49	±0.034	0.507
iso-16:0	0.37	±0.018	0.39	±0.019	0.587
16:0–2 Me	0.36	±0.028	0.46	±0.030	0.037
16:0	16.5	±0.41	14.9	±0.43	0.014
16:0–6 Me	0.51	±0.033	0.50	±0.035	0.761
16:0–8 Me	0.36	±0.021	0.40	±0.022	0.228
16:0–4 Me	0.68	±0.042	0.56	±0.044	0.051
16:0–12 Me	0.77	±0.040	0.74	±0.040	0.537
iso-17:0	0.48	±0.020	0.47	±0.021	0.654
16:1 *cis-*7	0.34	±0.016	0.38	±0.016	0.152
16:1 *cis-*9/ anteiso- 17:0	2.71	±0.101	2.74	±0.106	0.864
17:0	3.43	±0.120	3.47	±0.125	0.807
17:0–12 Me	0.23	±0.021	0.26	±0.022	0.393
iso-18:0	0.23	±0.013	0.23	±0.014	0.781
17:1 *cis-*9	1.98	±0.125	2.25	±0.121	0.163
18:0	13.0	±0.63	12.5	±0.66	0.613
18:1 *trans-*6/7/8	0.29	±0.009	0.28	±0.010	0.408
18:1 *trans-*9	0.22	±0.007	0.22	±0.007	0.588
18:1 *trans-*10	0.24	±0.016	0.25	±0.017	0.903
18:1 *trans-*11	1.59	±0.129	1.41	±0.135	0.361
18:1 *trans-*12	0.32	±0.022	0.32	±0.023	0.898
18:1 *cis-*9	37.1	±0.75	39.3	±0.79	0.063
18:1 *cis-*11	2.13	±0.086	2.16	±0.089	0.755
18:1 *cis-*12	0.55	±0.016	0.57	±0.017	0.314
18:1 *cis-*13	0.29	±0.025	0.27	±0.027	0.500
18:1 *trans-*16/ *cis-*14	0.47	±0.039	0.45	±0.041	0739
18:1 *cis-*15	0.12	±0.017	0.11	±0.019	0.521
18:2 *tt/ct/tc*	1.94	±0.127	1.86	±0.133	0.684
18:2 n-6	0.79	±0.040	0.69	±0.042	0.116
20:0	0.11	±0.005	0.12	±0.005	0.347
20:1 *cis-*11	0.04	±0.004	0.05	±0.005	0.060
18:3 n-3	1.03	±0.078	1.01	±0.081	0.812
18:2 *cis-*9, *trans-*11	0.90	±0.076	0.89	±0.079	0.930
18:3 *cis-*9, *trans-*11, *cis-*15	0.12	±0.008	0.08	±0.009	0.011
Other	1.06	±0.101	1.19	±0.105	0.373
Partial Sums					
iso + anteiso BCFA	1.96	±0.035	1.94	±0.068	0.852
non-terminal BCFA	6.90	±0.396	6.75	±0.413	0.794
Total BCFA	8.86	±0.425	8.69	±0.444	0.786
Odd linear FA	5.05	±0.167	5.11	±0.174	0.790
Total odd linear + BCFA	13.9	±0.53	13.8	±0.56	0.893
BI	7.01	±0.391	6.68	±0.409	0.568
Total C18 FA	61.1	±0.82	62.3	±0.86	0.302

Abbreviations: Me, methyl; BCFA, branched-chain fatty acids; BI, biohydrogenation intermediates, include total C18 FA minus 18:0, 18:1*cis-*9, 18:1*cis-*11, 18:2n-6 and 18:3n-3.

**Figure 2:**
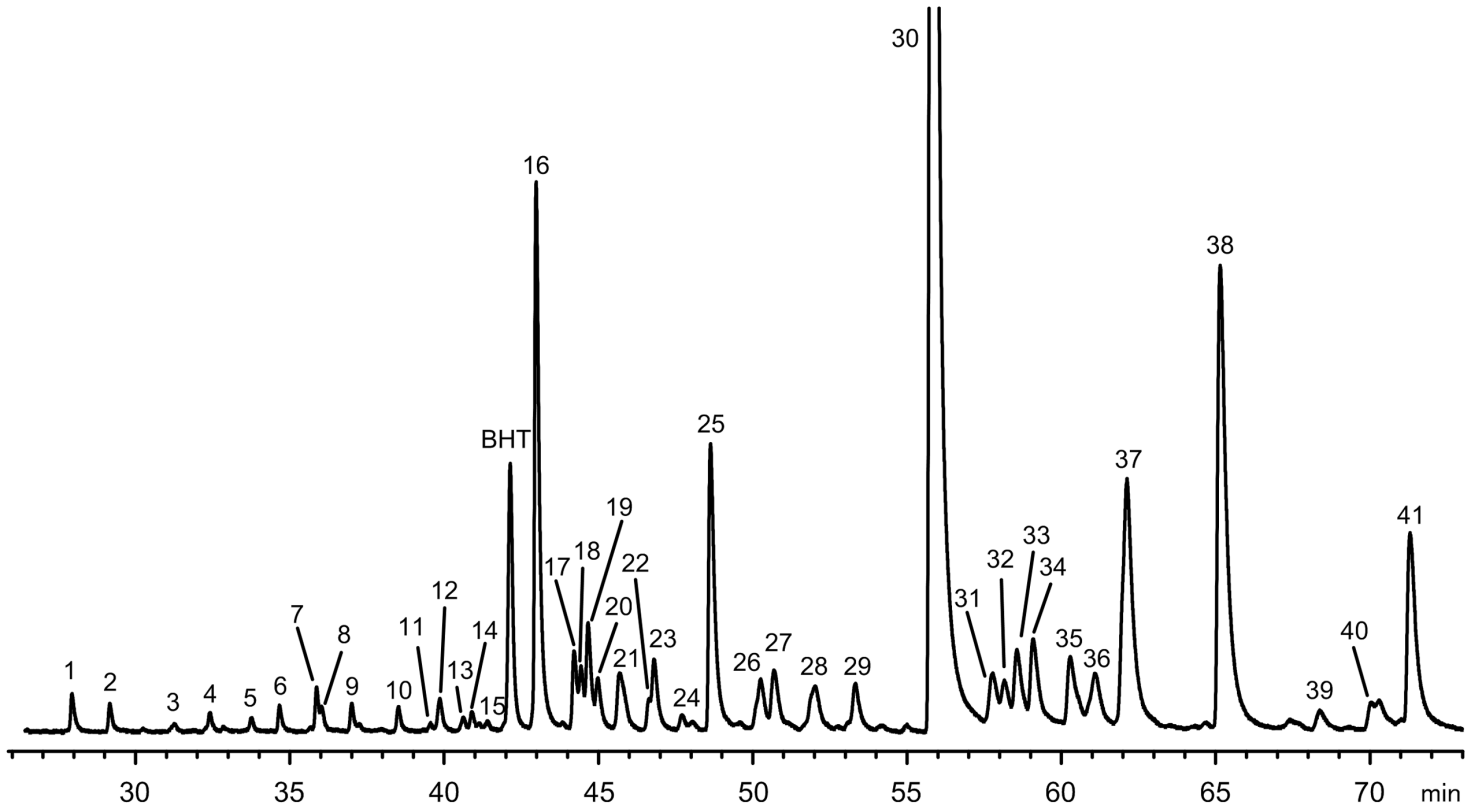
Partial gas-liquid chromatogram of the branched chain fatty acid region of the fat tail in Damara Ram lambs. Peak identification: 1) 10:0; 2) 10:0-4Me and 10:0–6Me; 3) 11:0; 4) 11:0–4Me; 5) 11:0–4,8Me; 6) 12:0; 7) 12:0–4Me; 8) 12:0–8Me; 9) 12:0–4,8Me; 10) 13:0; 11) 13:0–6Me; 12) 13:0–4Me; 13) i–14:0; 14) 13:0–4,8Me; 15) 14:0–2Me; 16) 14:0; 17) 14:0–6Me; 18) 14:0–8Me; 19) 14:0–4Me; 20) 14:0–10Me; 21) i-15:0; 22) 14:0–2,6Me; 23) a-15:0; 24) 14:1*cis-*9; 25) 15:0; 26) 15:0–8Me; 27) 15:0–4Me; 28) i-16:0; 29) 16:0–2Me; 30) 16:0; 31) 16:0–6Me; 32) 16:0–8Me; 33) 16:0–4Me; 34) 16:0–12Me; 35) i-17:0; 36) 16:1*cis-*7; 37) 16:1*cis-*9 and a-17:0; 38) 17:0; 39) 17:0–12Me; 40) i-18:0; 41) 17:1cis-9. Abbreviations: BTH, Butylated hydroxytoluene; Me, methyl; i, iso; a, anteiso.

**Figure 3:**
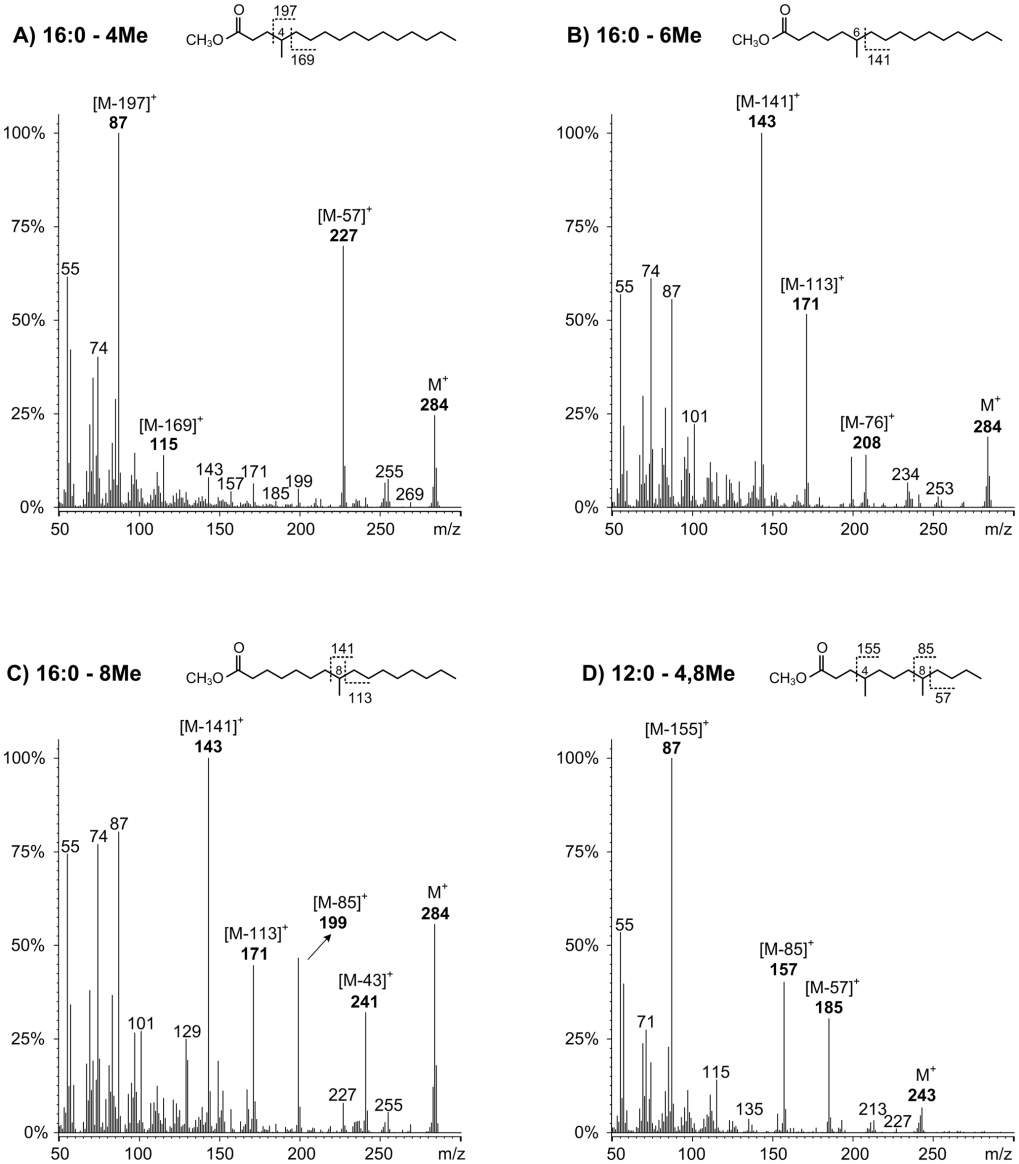
Electron impact mass spectra of the: A) 16:0–4Me; B) 16:0–6Me; C) 16:0–8Me; and D) 12:0–4,8Me branched chain fatty acids in the fat tail of Damara Ram lambs.

Figure 2 shows the elution profile of the BCFA in the fat tail. Most of these fatty acids are non-terminal mono-methyl BCFA with carbon chain lengths of 10 to 17 atoms. The identification by electron impact mass spectrometry revealed the presence of mono-methyl substituent in the position −2, −4, −6, −8, −10 and −12. The fragmentation of the mono-methyl BCFA was very similar to that described for the straight-chain fatty acids, with an abundant McLafferty rearrangement ion, carbomethoxy series ions, and hydrocarbon series fragment ions [11]. Although the position of the methyl substituent on the fatty acid carbon chain intensifies some fragment ions. Previous reports identified by mass spectrometry several non-terminal BCFA in the triacylglycerols of subcutaneous adipose tissues of lambs [12] and fallow deer [13]. These authors detected significant abundant ions at *m/z* 88 and *m/z* 87, corresponding to the 2-methyl and 4-methyl BCFA, respectively. Accordingly, the 2-methyl BCFA (14:0–2Me, 16:0–2Me) showed a major peak at *m/z* 88 due to McLafferty rearrangement and prominent carbomethoxy series ions at *m/z* 101, 157 and 159. The 4-methyl substituent was the most abundant mono-methyl branched; indeed, it was identified in fatty acids with carbon chain lengths from 11:0 to 16:0, accounting 2.4% and 2.2% of the total BCFA in growth and restricted animals, respectively. Their mass spectra showed intense carbomethoxy series ions at *m/z* 87 (base peak) and at M-57 (Figure 3-A). The 6-methyl substituent was detected in fatty acids with carbon chain lengths of 13:0, 14:0, and 16:0. Their mass spectra showed unique abundant ions at M-76 probably formed by a mechanism involving sequential loss of 32 and 44 mass units (Figure 3-B). The 8-methyl substituent was identified in the 14:0, 15:0 and 16:0 chain lengths by the intense ion at *m/z* 143 corresponding to cleavage of C7-C8 (Figure 3-C). Moreover, BCFA with methyl substituent at higher carbon chain length (-10Me and -12Me) were also identified in the 14:0, 16:0 and 17:0 fatty acids, showing intense ions at *m/z* 143 and 199, respectively.

Four di-methyl BCFA were tentatively identified in fat tail, from these the most abundant was the 4, 8-dimethyl substituent (11:0–4,8Me, 12:0–4,8Me, and 13:0–4,8Me). Their mass spectra showed intense ions at *m/z* 87 (base peak) and prominent ions at *m/z* 157 (corresponding to cleavage of C7–C8) and M-57 (Figure 3-D).

The fatty acid composition of the gastrocnemius muscle of Damara ram lambs is presented in Table 3. Total muscle fatty acid (mg/g DM) did not differ between treatments. Also, the muscle fatty acid profiles of animals submitted or not to feed restriction did not differ for the majority of the fatty acids, although the reduced number of observations and consequent lack of statistical power might contribute to that. Nevertheless, the fatty acids that differ significantly between groups were a few non-terminal BCFA and biohydrogenation intermediates. The biohydrogenation intermediates, 18:1 *trans-*6 to *trans-*10 and the *cis*-12, were lower in the muscle of animals submitted to feed restriction compared to animals allowed to grow. However, the proportion of the total biohydrogenation intermediates in muscle only showed a tendency (P = 0.079) to be higher in animals allowed to grow than in animals submitted to feed restriction.

**Table 3:** Effect of feed restriction in total fatty acid (mg/g DM) and dimethylacetal and fatty acid composition (% of total products) of the gastrocnemius muscle in Damara ram lambs.

Fatty Acid and DMA	Growth	SEM	Restricted	SEM	P value
N	5		7		
Total FA	29.2	±2.81	30.1	±2.37	0.820
12:0	0.10	±0.006	0.12	±0.005	0.065
iso-14:0	0.10	±0.009	0.10	±0.008	0.737
14:0	0.92	±0.145	1.01	±0.123	0.623
14:0–6Me	0.02	±0.008	0.03	±0.007	0.152
14:0-8Me + DMA-15:0	0.08	±0.015	0.09	±0.013	0.618
14:0–4Me	0.03	±0.014	0.06	±0.012	0.229
14:0–10Me	0.02	±0.006	0.03	±0.005	0.807
iso-15:0	0.06	±0.008	0.07	±0.007	0.421
anteiso-15:0	0.09	±0.012	0.12	±0.010	0.073
14:1 *cis-*9	0.45	±0.031	0.42	±0.026	0.443
15:0	0.22	±0.022	0.28	±0.018	0.052
DMA-16:0	3.58	±0.392	3.96	±0.331	0.474
iso-16:0	0.09	±0.004	0.10	±0.004	0.137
16:0	15.2	±0.82	13.8	±0.69	0.225
16:0–6 Me	0.05	±0.014	0.14	±0.012	<0.001
16:0–8 Me + DMA-17:0	0.32	±0.035	0.40	±0.029	0.090
16:0–4 Me	0.08	±0.021	0.24	±0.017	<0.001
16:0–12 Me	0.09	±0.023	0.23	±0.020	0.001
iso-17:0	0.40	±0.022	0.35	±0.018	0.047
16:1 *cis-*7	0.29	±0.021	0.28	±0.018	0.732
16:1 *cis-*9	0.93	±0.088	0.75	±0.074	0.155
anteiso-17:0	0.41	±0.033	0.40	±0.028	0.786
17:0	0.72	±0.052	0.85	±0.044	0.093
DMA-18:0	3.67	±0.405	3.90	±0.342	0.674
iso-18:0	0.09	±0.009	0.08	±0.008	0.288
17:1 *cis-*9	0.58	±0.071	0.73	±0.060	0.123
DMA-18:1	0.48	±0.042	0.55	±0.036	0.235
18:0	13.1	±0.47	13.9	±0.39	0.188
18:1 *trans-*6/7/8	0.11	±0.008	0.08	±0.007	0.014
18:1 *trans-*9	0.12	±0.005	0.11	±0.004	0.039
18:1 *trans-*10	0.21	±0.025	0.12	±0.021	0.033
18:1 *trans-*11	0.63	±0.080	0.69	±0.068	0.583
18:1 *trans-*12	0.11	±0.014	0.10	±0.012	0.578
18:1 *cis-*9	26.6	±1.70	24.3	±1.44	0.335
18:1 *cis-*11	1.97	±0.091	1.88	±0.077	0.450
18:1 *cis-*12	0.48	±0.020	0.41	±0.017	0.018
18:1 *cis-*13	0.09	±0.011	0.08	±0.009	0.247
18:1 *trans-*16/*cis-*14	0.18	±0.020	0.18	±0.017	0.947
18:1 *cis-*15	0.08	±0.012	0.08	±0.010	0.956
18:2 *tt/ct/tc*	0.74	±0.062	0.61	±0.053	0.131
18:2 n-6	11.8	±1.15	12.1	±0.98	0.844
19:1	0.22	±0.029	0.26	±0.024	0.350
20:0	0.08	±0.009	0.11	±0.008	0.037
20:1 *cis-*11	0.15	±0.008	0.15	±0.007	0.842
18:3 n-3	1.92	±0.220	2.49	±0.186	0.076
18:2 *cis-*9, *trans-*11	0.31	±0.036	0.30	±0.030	0.791
20:2 n-6	0.07	±0.009	0.06	±0.007	0.168
18:3*cis-*9, *trans-*11, *cis-*15^1^	0.79	±0.097	0.73	±0.082	0.670
22:0	0.42	±0.046	0.41	±0.039	0.928
20:4 n-6	4.29	±0.484	4.80	±0.409	0.444
20:5 n-3	1.81	±0.295	2.31	±0.249	0.231
22:5 n-3	1.98	±0.238	2.27	±0.201	0.379
22:6 n-3	0.80	±0.080	0.71	±0.067	0.413
Others	1.97	±0.114	1.69	±0.096	0.100
Partial Sums					
iso + anteiso BCFA	1.26	±0.062	1.21	±0.052	0.607
non-terminal BCFA	0.68	±0.094	1.21	±0.079	0.002
Total BCFA	1.85	±0.133	2.35	±0.113	0.017
Odd linear FA	0.94	±0.070	1.13	±0.059	0.064
Total odd linear + BCFA	2.79	±0.157	3.48	±0.186	0.018
BI	3.86	±0.146	3.49	±0.123	0.079
Total C18 FA	59.2	±0.85	58.2	±0.72	0.399

Abbreviations: Me, methyl; DMA, dimethylacetal; BCFA, branched-chain fatty acids; BI, biohydrogenation intermediates, include total C18 FA minus 18:0, 18:1 *cis-*9, 18:1 *cis-*11, 18:2 n-6 and 18:3 n-3. ^1^Co-elutes with the 20:3n-9.

**Figure 4:**
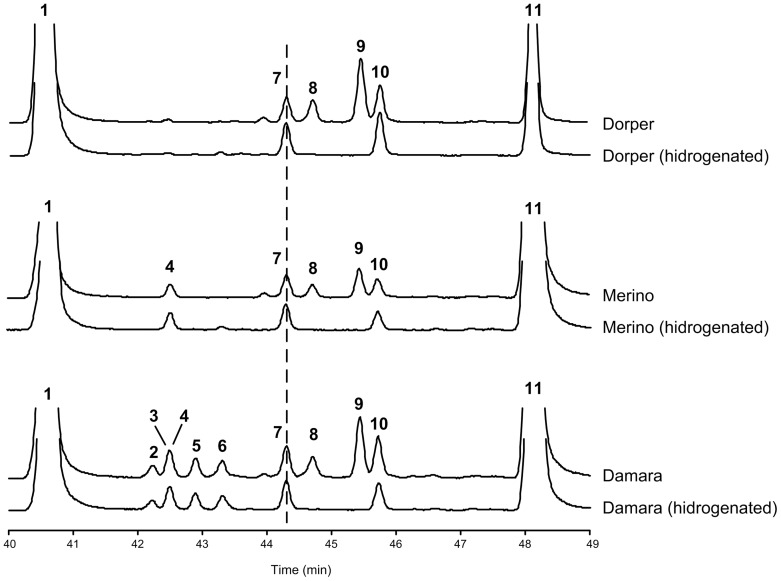
Partial gas-liquid chromatograms of the branched chain fatty acid region of gastrocnemius muscle samples of Damara, Merino and Dorper Ram lambs, before and after hydrogenation of the unsaturated fatty acids. Peak identification: 1) 16:0; 2) 16:0–6Me; 3) 16:0–8Me; 4) DMA-17:0; 5) 16:0–4Me; 6) 16:0–12Me; 7) i-17:0; 8) 16:1*cis-*7; 9) 16:1*cis-*9; 10) a-17:0; 11) 17:0.

Surprisingly, a few mono-methyl non-terminal BCFA of C14 and C16 linear chain lengths were present in the muscle of Damara ram lambs. The proportion of the total non-terminal BCFA was higher in animals submitted to feed restriction compared to animals allowed to grow (1.21 vs 0.68). However, only the C16 non-terminal BCFA differ between treatments (i.e. 16:0–6Me, 16:0–4Me and 16:0–12Me). The occurrence of these, non-terminal BCFA was checked in the gastrocnemius muscle of other breeds (Merino and Dorper) submitted to the same feeding regimens and trial. Interestingly, no non-terminal BCFA were detected in muscles from these two breeds. Muscle samples from the Damara, Merino and Dorper ram lambs were also catalytically hydrogenated to eliminate the unsaturated FA and render evident the BCFA. Hydrogenation confirmed the occurrence of the non-terminal BCFA in Damara muscle samples and the absence in Merino and Dorper muscle samples (Figure 4). Indeed, only the terminal iso and anteiso BCFA were detected in Merino and Dorper muscle. In Damara muscle, the iso and anteiso BCFA did not differ between feeding regimes, however the odd linear FA showed a tendency (P = 0.064) to be higher in restricted feed animals compared with those allowed to grow. Also, the proportion of total odd linear + BCFA was increased (P = 0.018) in restricted feed animals compared to animals allowed to grow (3.5 vs. 2.8% of total fatty acids).

## Discussion

Fat-tailed sheep breeds are widely recognized to be more tolerant to severe and prolonged undernutrition as they have the supplementary tail fat depot that serve as a steady but slow releasing source of fatty acids for the metabolism, as demonstrated in the Barbarine breed from North Africa [14]. In the present experiment, the reduction of tail fat depots in Damara submitted to feed restriction was proportional to live weight and carcass weight losses, as tail fat depot represent the 2.3% of live weight in both feed restricted and growth groups. This might be explained by the mild feed restriction (85% of maintenance requirements for 42 d) imposed to animals and by the fact that fat tail depot mobilization has a low priority order, after subcutaneous and perirenal fat depots [14].

Tail fat mobilization had therefore limited effects on the fatty acid profile suggesting that there is no differential metabolization of any particular fatty acid. Nevertheless, tail fat from feed restricted animals had 1.6 percent less 16:0 and tended to have (*P* = 0.063) 2.2 percent more 18:1 *cis-*9 than animals allowed to growth. Reduction in 16:0 might be explained by lower *de novo* lipogenesis in animals under feed restriction compared to the animals not submitted to feed restriction. The trend to have increased 18:1 *cis-*9 in tail fat of animals under feed restriction cannot be explained by the increased lipogenic and stearoyl-CoA desaturase activity and thus might be indicative that 18:1 *cis-*9 might be preferential retained during mobilization probably to ensure a low melting point for tail fat. In fact, reviewing published fatty acid profiles of tail fat depots, it is clear that this depot presents higher 18:1 *cis-*9 content than other fat depots [8], [14], [15]. Indeed, the unsaturated fatty acid, BCFA and odd-numbered fatty acids are recognized to largely contribute to lower lamb fat melting points [16]. Also, tail fats have lower melting points than internal fats [6]. In fact, it is known that the non-internal superficial depots have lower melting temperatures than internal fat depots [16], [17], and this might be more evident for the tail fat depot. In this context, the very high concentration of the BCFA found is perfectly consistent with the need to lower the melting point of tail fat depot, as this is recognized to reduce fat firmness in lambs [18]. However, to the best of our knowledge there are no previous reports of high concentrations of BCFA in adipose tissue from tail fat. Most reports only present the major fatty acids and it is not clear if BCFA were present but not identified and not reported, or if they were in fact absent.

The occurrence of high levels of BCFA in Damara tissues might be important due to the potential health effects in humans. Indeed, it was reported that iso and anteiso BCFA show antitumoral activity in human cancer cells [19], [20] and also reduce the incidence of necrotizing enterocolitis, a devastating intestinal disease affecting premature infants [21]. However, as far as we know, there is no information available about the potential health effects of the non-terminal mono- and di-methyl BCFA.

Nevertheless, the occurrence of such high concentrations of branched and odd chain fatty acids have been reported in unusually soft subcutaneous adipose tissue of fast growing lambs fed diets containing high concentrations of easily fermentable carbohydrates [18], [22]–[23]. Additionally, it has been proposed that these diets led to excessive propionate absorption that would exceed the liver gluconeogenesis resulting in increased propionate and methyl-malonate availability to adipose tissue. The ability of animal adipose tissue fatty acid synthases to use both propionate and methyl-malonate has long been demonstrated [24], [25]. Thus, *de novo* fatty acid synthesis in adipose tissue can use propionate as primer, leading to odd chain fatty acids, and methyl-malonate as elongation unit, leading to branched chain fatty acids. Elevated concentrations of odd and branched chain fatty acids also occur in cobalt and Vitamin B12 deficient lambs [26]. However, in the case of Damara animals both conditions are excluded. In fact, animals either in moderate growth or moderate feed restriction were equally fed only on roughage-based pellets with 9.3 MJ/kg DM of ME.

Direct comparisons of fatty acid composition of adipose tissue with the other two breeds were not possible because no adipose tissue samples other than Damara tail fat were collected. Thus, we analyzed muscle samples from the 3 breeds used in the experiment. As expected no branched chain fatty acids other than the common iso and anteiso fatty acids were detected in Dorper and Merino muscle samples. However, Damara muscle samples contained several mono-methyl non-terminal BCFA of C14 and C16 linear chain lengths, although at much lower concentration than in the tail fat tissue. We have a wide experience in analyzing the fatty acid composition of lamb meat and the occurrence of mono-methyl non-terminal BCFA is sporadic and always at trace amounts. Moreover, even in fast growing lambs with fairly high concentration of mono-methyl non-terminal BCFA (5.5% of total fatty acids) in dorsal adipose tissue had at most negligible (not reported) contents in meat [27]. So, the presence of these mono-methyl non-terminal BCFAs on Damara muscle samples is biologically relevant. The content of C16-Me FA (excluding the C16-8Me that co-elute with DMA-17:0) in muscle is about 11% than the content found in tail fat in animals allowed to grow and about 34% in animals under feed restriction. A similar pattern was observable in muscle for the non-terminal C14-Me BCFA, which was present at concentrations of about 5% and 9% of those found in tail fat, respectively for growth and restricted animals. Thus, animals submitted to feed restriction presented higher BCFA content particularly of non-terminal C16-Me than animals allowed to grow. As *de novo* FA synthesis is expected to be down regulated in feed restricted animals, the increased concentration of these non-terminal BCFA must reflect the increased mobilization of other fatty acids from the tail adipose deposit.

Overall, the data presented here, strongly suggests that the Damara breed presents unique lipid metabolism traits or regulation leading to elevated concentrations of odd and BCFA in tissues. The novelty is not due to the presence of very high content of odd and BCFA in adipose tissue, but the fact that in Damara animals, this occurs in spite of the fact that they were fed a roughage-based pellet and were under weight loss conditions. The fat tail adipose tissue was identified as putatively responsible for this metabolic peculiarity, being probably responsible for *de novo* synthesis of odd and BCFA and for supplying it to the muscle. However, it is not clear if this capacity is generalized to other adipose depots, particularly dorsal subcutaneous adipose tissue, or even if is restricted to the Damara unique long fat tail, different from other Southern Africa fat-tailed sheep breeds [28]. Additionally, it is not completely clear if other fat tail ovine breeds share this metabolic feature, although published fatty acid characterizations of their tail fat do not report the occurrence of these non-terminal BCFA.

We can only speculate on the mechanisms that determine this metabolic peculiarity of the Damara breed. As a determinant part of the Herero and Himba tribes subsistence farming systems, Damaras followed a fairly isolated selection in the semi-arid lands of Southern Africa, and suffered little or no influence from other (Southern) African or even European breeds, retaining therefore particularly important adaptive traits and possibly quite uncommon isoforms of key lipogenic enzymes. In several species, sebaceous glands produce a significant proportion of BCFA, and in some specialized glands like harderian, meibomian and uropygial glands, those BCFA comprise the majority of FA produced by fatty acid synthetase [29]. The high BCFA output of fatty acid synthetase in uropygial gland is generated by reducing the local availability of malonyl-CoA, due to the presence of highly active malonyl-CoA decarboxilase, resulting in proportional increased availability of methylmalonyl-CoA formed by propionate carboxylation, as a chain elongation substrate [30]. Damara tail fat may therefore present similar local metabolic control involving one or more enzymes linked to propionate metabolism or malonyl catabolism.

Nevertheless, the similarity of odd and BCFA profiles found in Damara tail fat with dorsal fat of fast growing lambs overloaded with propionate is puzzling, considering that Damara animals were fed a roughage-based feed. Recently, Wilkes et al. [31] reported that Damara have a higher capacity to obtain more nutrients from low-quality diet than Merino sheep. This suggests that Damara sheep have also digestive peculiarities probably linked to rumen kinetics and volume and eventually rumen fermentation. Efficient digestion of high fibrous diets is known to lead to a high acetate and low propionate formation in the rumen [32]. Thus, being adapted to low quality fibrous diet, it is possible that Damara breed also presents a lower threshold for propionate metabolism. Therefore, more precursors might be available to the tail fat, due to an eventual lower rate of hepatic metabolism.

Thus, in order to clarify the apparent metabolic peculiarity of the Damara animals, the occurrence of uncommon isoforms and gene expression patterns of key lipogenic enzymes in Damara tissues, particularly on fat depots, must be investigated. Moreover, propionate and methyl-malonyl metabolism need to be evaluated in Damara animals subjected to diets differing in fiber/starch content.
